# Supplementary material for: Occult hepatitis B virus infections and risk factors among school-going adolescent voluntary blood donors in Kwale County Kenya, January 2020–June 2021: Cross sectional study
Source: PLoS One. 2022 Jul 25;17(7):e0263473. doi: 10.1371/journal.pone.0263473 (PMC9312369; doi:10.1371/journal.pone.0263473)
Supplement: Attachment — Submitted filename: Response to Reviewers_HBV_Manuscript.docx [file pone.0263473.s002.docx]

Dear reviewer # 1 Thank you for reading and reviewing the manuscript titled “***Occult Hepatitis B virus infection and risk factors Among School-going Adolescent Voluntary Blood Donors in Kwale County, Kenya, January 2020–June 2021*** ***:*** **Cross sectional study”** and seeing it is scientifically written and interesting. Find below tables with corrections and answers which can also be found in the Manuscript as highlighted.

| Serial number | Comments /questions | Corrections/ responses |
| --- | --- | --- |
| Concern 1 | The introduction is too long and the purpose is not clear, please rewrite it clearly; | The introduction part has been revised and clearly written and shortened as requested by the reviewer |
| Concern 2 | The implication of the occult HBV infection is not clear neither in the introduction nor in the discussion sections; | - The implication of OBI has been captured in lines 60-70 then lines 74-78 see below. - A person with occult hepatitis B infection (OBI) has a risk of reactivation during immunosuppression and due to lack of detectable HBsAg there is an ongoing risk of transmission to others [4]. - A majority of people are unaware of their HBV infection, and often present with advanced disease [13], as noted in Nigeria where 5.4% of the donors had anti-HBcIg M as the only serological evidence of HBV infection [14]. - In Africa, the prevalence of OBI is estimated to be between 7 - 50% among blood donors or healthcare workers [6, 7] and 6 - 30% in HIV infected populations [8]. High rates of OBI was reported in HIV-positive individuals initiating antiretroviral therapy in Botswana [9]. - In sub-Saharan Africa, 12.5% of patients who receive blood transfusion are at risk of post-transfusion OBI [10]. - In Kwale County, while it is known that the annual demand for blood is approximately 9000 units according to the 2019 census population [12], no such study has been conducted in this area and therefore the prevalence of OBI is not known. |
| Concern 3 | Row 62: Reference 5 is too old, please update the knowledge about HBV genotypes and serotypes (there are 10 genotypes not 8); | .  The reference has been removed |
| Concern 4 | . Row 169: include a space between comprised and 335 | It has been corrected see line 151 comprised 335 individuals |
| Concern 5 | Row 179: « females » instead of « females’ » | It has been corrected see line 165  females comprised10.9% (17/156), |
| Concern 6 | Row 188: 18-25 age groupe and not 18-15; | It has been corrected see line 177  more frequently compared to 18-25 age group, |
| Concern 7 | Row 225: « male donors » instead of « male’s »; | It has been corrected see line 227-228, Compared to male donors, |
| Concern 8 | Row 237: « level of education; hence, our results » instead of « level of education hence our results » | It has been corrected see line 239 attending secondary level of education; hence, our results may |
| Concern 9 | Row 245: the majority … is male, is blood group O positive not are blood group O positive | voluntary young blood donors were male, is blood group O positive, |
